# Draft genome of the endophytic *Bacillus velezensis* CNPMS-22 isolated from maize leaves

**DOI:** 10.1128/mra.01279-24

**Published:** 2025-05-07

**Authors:** José Edson Fontes Figueiredo, Mikaely Sousa Marins, Felipe Campos Silva, Vitória Palhares Ribeiro, Gisele de Fátima Dias Diniz, Victor Alef Rodrigues, Christiane Abreu Oliveira-Paiva, Ubiraci Gomes de Paula Lana, Lourenço Vitor Silva Ferreira, Sylvia Morais de Sousa Tinoco, Valter Cruz-Magalhães

**Affiliations:** 1 Biochemistry Molecular Laboratory, Embrapa Maize and Sorghum, Sete Lagoas, Minas Gerais, Brazil; 2 Soil Microbiology Laboratory, Embrapa Maize and Sorghum, Sete Lagoas, Minas Gerais, Brazil; 3 Microbiology Laboratory, Universidade Federal de Viçosa, Viçosa, Minas Gerais, Brazil; 4 Soil Microbiology Laboratory, Universidade Federal de São João del Rei-UFSJ-CSL, Sete Lagoas, Minas Gerais, Brazil; 5 Núcleo de Biologia Aplicada, Embrapa Maize and Sorghum, Sete Lagoas, Minas Gerais, Brazil; 6 Microbial Ecology Laboratory, Embrapa Maize and Sorghum, Sete Lagoas, Minas Gerais, Brazil; University of Strathclyde, Glasgow, United Kingdom

**Keywords:** genome analysis, antimicrobial activity, plant growth promoter, endophytes

## Abstract

*Bacillus velezensis* has been widely used as a biocontrol agent and plant growth promoter in agricultural bio-inputs. The genome of the endophytic bacterial strain CNPMS-22 isolated from maize leaves was sequenced, and the results showed that the strain is a *Bacillus velezensis* species.

## ANNOUNCEMENT

*Bacillus velezensis* is a ubiquitous species isolated from diverse niches (1). It exhibits biocontrol activity against fungi, insects, and nematodes and promotes plant growth (2, 3). The endophytic strain CNPMS-22 was isolated in 2007 from maize leaves collected from the experimental field of the Embrapa Maize and Sorghum Research Center (19°26′43.6″ S, 44°10′03.0″ W) located in Sete Lagoas, Brazil, following a protocol for microbial isolation from plants (4). The bacterium was cryo-preserved at −80°C in a glycerol-based medium and deposited in the Embrapa Collection of Multifunctional and Phytopathogenic Microorganisms of Maize and Sorghum.

For genome sequencing, an aliquot of the glycerol stock was streaked onto a plate containing the Luria-Bertani (LB) agar medium and incubated for 48 h at 28°C. A pure colony recovered from the plate was inoculated into a 5 mL liquid LB medium and incubated for 48 h at 28°C. The genomic DNA was extracted using the Wizard Genomic DNA Purification Kit (Promega, USA) and quantified on the Qubit 2.0 fluorometer (Thermo Fisher Scientific Inc. [NYSE: TMO], MA, USA). According to the manufacturer’s recommendations, the library was prepared with a Nextera XT kit (Illumina, San Diego, CA, USA). Library quality was checked in GelBot (Loccus, Cotia, SP, Brazil). GoGenetic Company (Curitiba, PR, Brazil) sequenced the genome using the Illumina NextSeq 1000 platform with a P1-600 kit in a paired-end strategy. The Trimmomatic version 0.395 software (5) was used for quality control. Sequencing of the CNPMS-22 genome 5,005,400 reads, each 300 bp long, which were used for genome assembly by SPAdes version 3.12.0 (6). The assembly quality was assessed using Quast version 5.0.2 (7), and the completeness was evaluated using Benchmarking Universal Single-Copy Orthologs (version 5.3.1) (8) with the *bacteria_odb10* lineage data set (*n* = 124 bacterial orthologs). The NCBI Prokaryotic Genome Annotation Pipeline version 6.8 (9) performed the draft genome annotation.

The CNPMS-22 genome sequencing generated 5,005,400 paired-end reads, totaling ≈1.5 gigabytes of data and a theoretical coverage ≈373×. After quality filtering, the assembly generated 123 contigs with an *N*_50_ value of 258,002 and a final coverage genome equal to 8.0× across retained contigs. The genome has a length of 4,040,390 bp and 4,038 genes for 3,813 proteins, 84 tRNAs, 14 rRNAs, 5 ncRNAs, 1 tmRNA, and 46.5% G + C content. To confirm the taxonomic position of CNPMS-22, we used the fastANI program (10) and the Type Strain Genome Server (version 39.1) (11) for digital DNA-DNA hybridization (dDDH). The average nucleotide identity (ANI) value and taxonomic index dDDH were calculated concerning the type strain *B. velezensis* FZB42 (GenBank accession number NC_009725.2) (12). The ANI values and DDH index were 98.67% and 96.4%, respectively, which confirmed that CNPMS-22 is phylogenetically related to the type strain FZB42. Default parameters were used for all software.

Genome analysis and manual genome mining identified the three gene clusters predicted to synthesize iturin, surfactin, and fengycin, corroborating our previous ultra-performance liquid chromatography-mass spectrometry studies with CNPMS-22 (13). We also found that CNPMS-22 harbors other non-ribosomal, ribosomal, and polyketide genes predicted to encode for molecules with antifungal activity.

## Data Availability

The draft genome sequence of *B. velezensis* strain CNPMS-22 has been deposited in GenBank BioProject PRJNA1108656, BioSample SAMN43568027, accession number JBHGBV000000000.1, contig-level assemblies JBHGBV01, NCBI RefSeq assembly GCF_041949825.1, and Sequence Read Archive (SRA) SRR31156977.
